# Gastric Mucosa Infection Caused by Sarcina Associated With Ulceration and Upper Gastrointestinal Bleeding

**DOI:** 10.7759/cureus.77871

**Published:** 2025-01-23

**Authors:** Carolina R Lima, Abadia Matoso, Camila R Tibiletti, Roberta Kazan, Bruno C Dornelas

**Affiliations:** 1 Medicine, Federal University of Uberlândia, Uberlândia, BRA; 2 Gastroenterology and Hepatology, Clinical Hospital of Federal University of Uberlândia, Uberlândia, BRA; 3 Gastroenterology, Clinical Hospital of Federal University of Uberlândia, Uberlândia, BRA; 4 Cytopathology, Instituto Master de Ensino Presidente Antônio Carlos (IMEPAC) - Centro Universitário, Araguari, BRA; 5 Pathology, Clinical Hospital of Federal University of Uberlândia, Uberlândia, BRA

**Keywords:** clinical infectious medicine, sarcina, sarcina species, stomach ulcers, upper gastrointestinal(ugi) bleeding

## Abstract

*Sarcina sp. (Ss) *infection is a rare cause of gastrointestinal disease, with only 66 cases reported worldwide. We present a case of a 56-year-old man with alcohol abuse, smoking, and chronic obstructive pulmonary disease who presented with upper gastrointestinal bleeding and decompensated cirrhosis. Endoscopy and biopsy revealed gastric ulcers with tetrad-shaped cocci consistent with *Ss*. After 32 days of hospitalization and broad-spectrum antibiotic therapy, both clinical and endoscopic improvement was observed. This case highlights the atypical presentation of this increasingly recognized infection and the importance of prompt diagnosis and supportive care.

## Introduction

*Sarcina *sp.* *(*Ss*) is an anaerobic, gram-positive, coagulase-negative bacterium with fermentative carbohydrate metabolism, capable of surviving in extremely low pH environments and it occurs in tetrads or packets of eight or more cells resulting from division in perpendicular planes [1]. It was first described by the Scottish anatomist and biologist John Goodsir in 1842 after a microscopic analysis of the gastric contents of a patient with daily vomiting [1]. Its morphological characteristics include an almost spherical shape, with individual sizes ranging from 1.8 to 3μm, a refractory nature, basophilic staining to hematoxylin-eosin (HE), and the presence of extracellular cellulose [2]. The bacterium's natural habitat is soil, but it is also found in water and air as spores [3]. Transmission occurs orally through contaminated water and food [1-4]. The gastrointestinal tract, predominantly the stomach, is the most common site of infection, although it has also been detected in the blood, lungs and urine [4-6].

A total of 66 cases have been documented in the medical literature, with a notable increase in reports since 2010 [1,3,7]. Abdominal pain, dyspepsia, nausea, vomiting and bloating are the main clinical symptoms [2,7,8]. However, a multitude of additional symptoms have been documented, including melena, hematemesis, weight loss, emphysematous gastritis, gastric perforation, anemia and iron deficiency, as well as hemodynamic instability and death [1-14].

The primary risk factors encompass a medical history marked by prior gastrointestinal surgery, notably bariatric surgery, along with conditions such as diabetes mellitus, hypertension, hypothyroidism, chronic liver disease, and associated infections such as *Helicobacter pylori*, *Giardia lamblia* and hepatitis [4, 8,15-17]. Additionally, conditions that result in delayed gastric emptying, including stenosis and obstruction, are considered risk factors [4,17].

Very little is known about *Ss* infection. We present a case of a patient with a gastric ulcer exhibiting malignant characteristics, complicated by upper gastrointestinal bleeding attributed to* Ss* infection. This case was previously presented as a poster at the Brazilian Digestive System Week on November 23, 2023.

## Case presentation

A 56-year-old male patient with a documented history of alcohol abuse and smoking presented with acute symptoms including hematemesis, persistent nausea, and epigastric pain. He sought medical attention the following day, at which time he exhibited a recurrence of hematemesis, melena, high fever (>38.3ºC), and abdominal distension. The initial examination revealed pale mucous membranes and signs of dehydration. Vital signs indicated a heart rate of 80 beats per minute, an oxygen saturation of 86% (without respiratory distress), and a blood pressure of 84/60 mmHg. On physical examination, the abdomen was distended and tender. He also had lower extremity edema and presented moderate ascites, sarcopenia, and grade 1 encephalopathy.

Laboratory findings (Table 1) revealed mild anemia, leukocytosis, and a concomitant slight increase in transaminase levels. Urine analysis was unremarkable, and chest X-ray and echocardiogram results were normal. Paracentesis revealed spontaneous bacterial peritonitis. Serological testing for HIV, hepatitis, and syphilis was negative, as were tests for *Clostridioides difficile* toxins A and B.

**Table 1:** Baseline Laboratory Data ALT: alanine aminotransferase; AST: aspartate aminotransferase

Test	Result	Reference Ranges
Hemogram	-	-
Red blood cells	2.49 million/mm³	4.30-5.70 million/mm³
Hemoglobin	8.9 g/dL	13.5-17.5 g/dL
Hematocrit	24.9%	39-50%
Platelets	165,000/mm³	150-450,000/mm³
Leukocytes	12.49 mil/mm³	3.5-10.5 mil/mm³
Arterial gasometry	-	-
pH	7.41	7.35-7.45
pCO2	33.6 mmHg	35-48 mmHg
PO2	44 mmHg	83-108 mmHg
HCO3	21.3 mmol/L	21-28 mmol/L
Lactate	4.28 mmol/L	0.36-0.75 mmol/L
Biochemistry tests	-	-
Gamma-glutamyl transferase	146 U/L	< 55 U/L
ALT	55 U/L	11-34 U/L
AST	15 U/L	< 45 U/L
Total bilirubin	3.5 mg/dl	0.2-1.2 mg/dL
Direct bilirubin	2.56 mg/dl	< 0.5 mg/dL
Indirect bilirubin	0.94 mg/dl	< 0.7 mg/dL
Urea	69 mg/dL	18-55 mg/dL
Creatinine	1.92 mg/dL	0.7-1.25 mg/dL
Sodium	134 mmol/L	135-145 mmol/L
Potassium	3.4 mmol/L	3.5-4.5 mmol/L
Albumin	2 g/dL	3.5-5.0 g/dL
C-reactive protein	12.41 mg/dL	< 0.5 mg/dL
Paracentesis	-	-
Protein	1.4 g%	-
Albumin	0.6 g%	-
Cellularity (polymorphonuclear cells)	2,900/mm³ (80%)	-
Red blood cells	1,605/mm³	-
Bacterial culture	Negative	-

Esophagogastroduodenoscopy (EGD) revealed Los Angeles grade C erosive esophagitis with four medium-caliber varicose veins, but without evidence of recent bleeding. Gastric examination showed edema and varied mucosal changes throughout the body, fundus, and antrum. A 1.5-cm irregular, raised, fibrin-covered lesion with erythematous edges and central necrosis was present in the greater curvature, suggesting malignancy. The antrum demonstrated well-demarcated edema with multiple flat erosions, consistent with portal hypertensive gastropathy and antral flat erosive gastritis.

**Figure 1:**
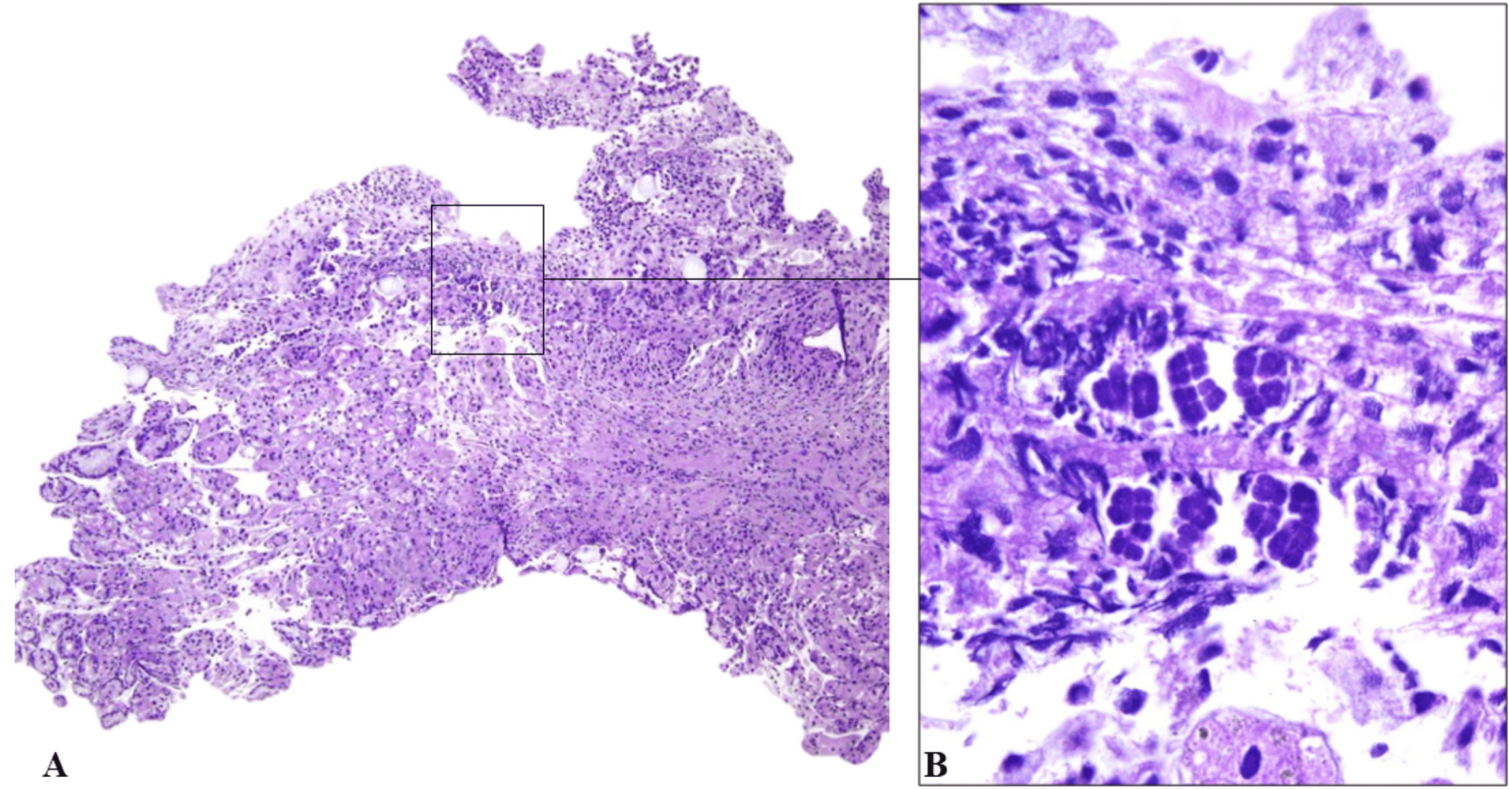
Histopathological examination of gastric ulcer tissue (A) Low-power view (HE stain, 10x magnification) showing a section of oxyntic mucosa exhibiting an area of acute ulceration. (B) High-power view (HE stain, 100x magnification) illustrating numerous gram-positive cocci arranged in characteristic cuboidal tetrads, consistent with *Sarcina* sp.

A biopsy of the greater curvature of the gastric body, where the ulcer was located, revealed acute erosive gastritis with stigmata of portal gastropathy. However, metaplasia, dysplasia, or malignancy was not observed. *Helicobacter pylori* testing was negative. Microscopic examination, via routine HE staining, showed tetrahedral cocci consistent with *Ss* adhering to the gastric mucosa (Figure 1). Initial treatment included omeprazole (40 mg), metronidazole (750 mg) for 10 days, ceftriaxone (2 g) for 16 days, oxacillin (1 g) for 14 days, and piperacillin/tazobactam (9 g/1,125 g) for 10 days.

A repeat EGD on day 26 showed a 1.5-cm irregular ulcer in the greater curvature with a narrow base, thin fibrin, and scar retraction. The antrum displayed multiple telangiectasias and flat erosions, consistent with antral vascular ectasia. During the 32-day hospitalization due to hematemesis and decompensated cirrhosis, no further gastrointestinal bleeding occurred.

Three months after being discharged from the hospital, the patient underwent a follow-up endoscopy that revealed an antral pseudodiverticulum, likely secondary to ulcer healing. Notably, the patient did not experience any further gastrointestinal bleeding and remained asymptomatic. Prophylactic treatment consisted of endoscopic rubber band ligation of esophageal varices, ciprofloxacin (500 mg), and omeprazole (80 mg) to prevent bacterial peritonitis.

## Discussion

The precise pathogenesis of *Ss* infection in humans remains unclear, though it is hypothesized that the bacterium damages the gastrointestinal mucosa by accumulating metabolic byproducts of carbohydrate fermentation, including acetaldehyde, hydrogen, carbon dioxide, ethanol, lactic acid, and acetone [2]. This, combined with conditions promoting bacterial growth and mucosal fragility, likely increases pathogenicity [8]. Biopsy findings frequently reveal a range of conditions, including gastric outlet obstruction, gastroparesis, concomitant infections, and chronic diseases associated with bacterial overgrowth [1-4]. Gastric stasis may further exacerbate the infection by increasing the availability of carbohydrate substrates, thereby enhancing bacterial proliferation [4, 8].

Clinical manifestations are highly variable, ranging from asymptomatic cases to life-threatening complications such as hemodynamic instability secondary to gastric perforation [2, 14, 18]. Symptoms may include abdominal pain, nausea, vomiting, and distension [3, 8, 9]. While *Ss.* infection has been reported across all age groups (1-87 years, mean 37.4 years), it appears more prevalent in females (2:1 ratio) [1, 4, 5]. Our patient was older than the typical age at presentation, and presented with abdominal pain, nausea, vomiting, and upper gastrointestinal bleeding. Pre-existing conditions such as chronic lung and liver disease and alcohol abuse, which can alter gastric motility and increase mucosal fragility [19], likely contributed to the severity of his manifestation. EGD findings, however, revealed a lesion mimicking gastric cancer. Common endoscopic findings in *Ss* infection include retained food debris and epithelial barrier disruption (erosion, ulceration, necrosis, stenosis) [4, 8].

The distinctive histologic features of *Ss* enable diagnosis via routine HE staining [1]. Molecular methods such as 16S rRNA polymerase chain reaction or pyruvate decarboxylase gene PCR can confirm the diagnosis [8]. It is important to differentiate *Ss* from other bacteria exhibiting tetrad arrangements, particularly Micrococcus spp., which are aerobic, catalase-positive, and do not form spores [2].

Treatment guidelines remain inconsistent, with no established standard regimen or duration [2]. In our case, broad-spectrum antibiotic therapy was initiated due to the ulcer, erythema, and bacterial growth. The patient showed significant endoscopic improvement and resolution of symptoms following the treatment completion. While antibiotic therapy is not always necessary in asymptomatic individuals, early antibiotic treatment is crucial for preventing complications and reducing mortality in cases with severe disease [19].

Antimicrobial selection is hampered by limited data on efficacy and standard regimens [16]. Antibiotic therapy is recommended for patients with gastric lesions to mitigate the risk of severe complications, such as perforation [2]. Metronidazole combined with other antibiotics is often used, with careful monitoring of treatment efficacy [5]. Regimens may include metronidazole alone, clarithromycin, gentamicin, vancomycin, imipenem, amoxicillin, fluoroquinolone, fluconazole, or amphotericin B [1-4]. In cases of mild symptoms without visible gastric lesions, proton pump inhibitors and prokinetics may be sufficient, with follow-up endoscopy recommended [16]. In rare instances, surgical intervention (gastrectomy, jejunostomy, ileocecal or duodenal resection) may be necessary [9]. Intermittent fasting may also be considered as an adjunctive measure to reduce carbohydrate substrates available to the bacteria [2].

## Conclusions

While *Ss* infection remains rare, its identification has increased in recent years. This case report highlights the importance of increased awareness of its clinical manifestations and the potential for improved prognosis with early treatment. Unlike typical cases, this patient's ulcer, which healed completely following broad-spectrum antibiotic therapy, was not attributable to *Helicobacter pylori* infection, chronic non-steroidal anti-inflammatory drug use, or other identified causes. In patients presenting with idiopathic gastric ulcers, particularly those with chronic conditions and risk factors such as gastroparesis or prior gastric surgery, *Ss* should be considered in the differential diagnosis. Further research is needed to elucidate the pathogenesis of this infection and to determine optimal treatment strategies, given the current paucity of data and the rarity of reported cases.
